# Correction: Covalent engineering of a phenanthroline-modified NH_2_-MIL-53(Al) MOF for the dual-mode sensing of As^3+^ and Fe^2+^ in complex environmental and dietary matrices

**DOI:** 10.1039/d6ra90062c

**Published:** 2026-07-06

**Authors:** Salhah D. Al-Qahtani, Ghadah M. Al-Senani, Abeer Abdulaziz H. Bukhari, Menier Al-Anazi, Humaira Parveen, Uzma Faridi, M. A. M. El-Afify

**Affiliations:** a Department of Chemistry, College of Science, Princess Nourah Bint Abdulrahman University P.O. Box 84428 Riyadh 11671 Saudi Arabia; b Department of Chemistry, Faculty of Science, University of Tabuk Tabuk 71491 Saudi Arabia; c Department of Biochemistry, Faculty of Science, University of Tabuk Tabuk Saudi Arabia; d Egyptian Propylene and Polypropylene Company Port Said 42511 Egypt maher.elafify@yahoo.com

## Abstract

Correction for ‘Covalent engineering of a phenanthroline-modified NH_2_-MIL-53(Al) MOF for the dual-mode sensing of As^3+^ and Fe^2+^ in complex environmental and dietary matrices’ by Salhah D. Al-Qahtani *et al.*, *RSC Adv.*, 2026, **16**, 27718–27737, https://doi.org/10.1039/D6RA02963A.

The authors regret that in the published version of this article, Fig. 3B was unintentionally duplicated due to a copy and paste error. The authors have provided the correct Fig. 3B and C below.

**Fig. 1:**
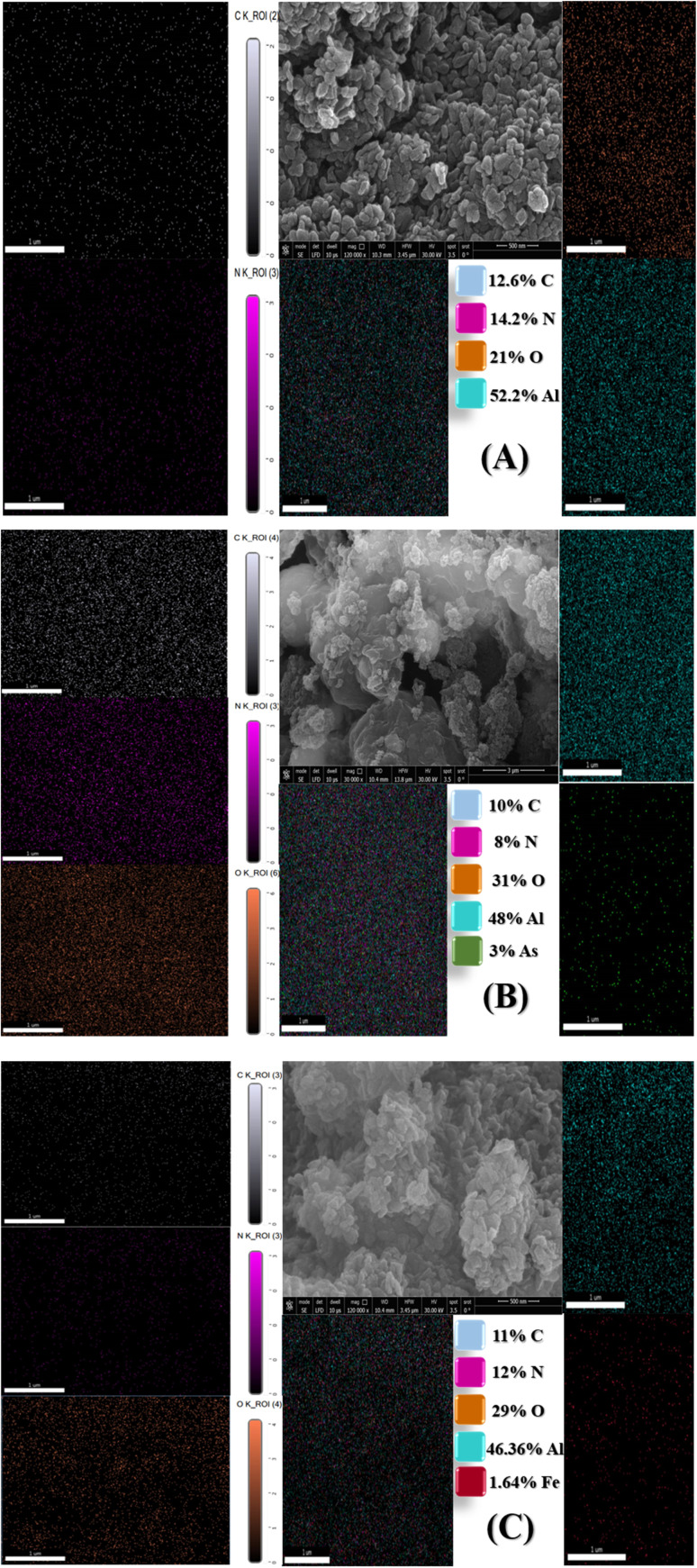
(A) SEM mapping analysis of Phen-GA-MIL-53(Al) sensor, (B) As^3+^@Phen-GA-MIL-53(Al) complex, and (C) Fe^2+^@Phen-GA-MIL-53(Al) complex.

The Royal Society of Chemistry apologises for these errors and any consequent inconvenience to authors and readers.

